# Neural stem cell-derived small extracellular vesicles attenuate apoptosis and neuroinflammation after traumatic spinal cord injury by activating autophagy

**DOI:** 10.1038/s41419-019-1571-8

**Published:** 2019-04-18

**Authors:** Yuluo Rong, Wei Liu, Jiaxing Wang, Jin Fan, Yongjun Luo, Linwei Li, Fanqi Kong, Jian Chen, Pengyu Tang, Weihua Cai

**Affiliations:** 1 Department of Orthopaedics, First Affiliated Hospital of Nanjing Medical University, Nanjing, 210029 Jiangsu China; 2 Department of Orthopaedics, West China Hospital Sichuan University, Chengdu, 610000 Sichuan China

**Keywords:** Cell death in the nervous system, Neural stem cells, Neural stem cells, Neural stem cells, Neural stem cells

## Abstract

Spinal cord injury (SCI) can cause severe irreversible motor dysfunction and even death. Neural stem cell (NSC) transplantation can promote functional recovery after acute SCI in experimental animals, but numerous issues, including low-transplanted cell survival rate, cell de-differentiation, and tumor formation need to be resolved before routine clinical application is feasible. Recent studies have shown that transplanted stem cells facilitate regeneration through release of paracrine factors. Small extracellular vesicles (sEVs), the smallest known membrane-bound nanovesicles, are involved in complex intercellular communication systems and are an important vehicle for paracrine delivery of therapeutic agents. However, the application of NSC-derived small extracellular vesicles (NSC-sEVs) to SCI treatment has not been reported. We demonstrate that NSC-sEVs can significantly reduce the extent of SCI, improve functional recovery, and reduce neuronal apoptosis, microglia activation, and neuroinflammation in rats. Furthermore, our study suggests that NSC-sEVs can regulate apoptosis and inflammatory processes by inducing autophagy. In brief, NSC-sEVs increased the expression of the autophagy marker proteins LC3B and beclin-1, and promoted autophagosome formation. Following NSC-sEV infusion, the SCI area was significantly reduced, and the expression levels of the proapoptotic protein Bax, the apoptosis effector cleaved caspase-3, and the pro-inflammatory cytokines TNF-α, IL-1β, and IL-6 were significantly reduced, whereas the expression level of the anti-apoptotic protein Bcl-2 was upregulated. In the presence of the autophagy inhibitor 3MA, however, these inhibitory effects of NSC-sEVs on apoptosis and neuroinflammation were significantly reversed. Our results show for the first time that NSC-sEV treatment has the potential to reduce neuronal apoptosis, inhibit neuroinflammation, and promote functional recovery in SCI model rats at an early stage by promoting autophagy.

## Introduction

Spinal cord injury (SCI) often leads to severe dysfunction of the limbs below the injured spinal segment, and more rostral (cervical injuries) may even be fatal^1^. The ultimate severity of SCI depends both on the initial trauma, which physically destroys neurons or severs axons, and on the extent of delayed secondary damage owing to inflammation, which can cause edema, neuronal apoptosis, cavitation, and reactive gliosis^2,3^. The treatment of SCI is rapidly evolving, and some experimental treatments have been examined in clinical trials. However, SCI remains largely irreversible and it is currently uncertain whether these therapies can safely improve prognosis^4^.

Cell transplantation is one promising strategy to improve the recovery of motor, sensory, and/or autonomic function after SCI^5,6^. Neural stem cells (NSCs) have the ability to self-renew and produce neurons, oligodendrocytes, and astrocytes^7–9^. In recent years, NSC transplantation has become a major focus in the study of SCI repair^10^. Indeed, several studies have shown that NSC transplantation has a unique neuroprotective function that promotes functional recovery after acute SCI. The beneficial effects of NSC transplantation are mediated primarily by the promotion of regeneration, plasticity, and neurogenesis, and by the suppression of neuroinflammation^11–13^. However, direct transplantation of stem cells into target tissues remains challenging. For example, transplanted stem cells have a low survival rate owing to ischemia^14^. Other risks, such as cell de-differentiation, immune rejection, and tumor formation, further limit the clinical application of direct stem cell transplantation for the treatment of SCI^15,16^.

Recent studies have shown that transplanted stem cells confer neuroprotection and promote repair primarily through a paracrine mechanism, and small extracellular vesicles (sEVs) play an important role in this process^17^. sEVs are the smallest endocytic membrane-bound nanovesicles^18,19^. They are released from many cell types under normal or pathological conditions and affect the activity of the recipient cells by delivery of various bioactive signaling molecules. sEVs contain cellular proteins and lipids involved in rapid signaling transduction as well as a host of functional mRNAs and microRNAs^20^. As stem cell secretions appear to be more beneficial for tissue regeneration and repair than living stem cells per se, NSC-derived small extracellular vesicles (NSC-sEVs) have been examined for therapeutic effects^21^. sEVs are enriched in specific microRNAs that regulate multiple functions under physiological and pathological conditions, including modulation of the direct microenvironment, promotion of viral entry into cells, metabolic signaling, and regulation of diverse aspects of brain function in adulthood including the process of aging^22–25^. In addition, NSC-derived extracellular vesicles are used to treat a variety of neurodegenerative diseases owing to their anti-inflammatory, neurogenic, and neurotrophic effects^26–28^. Therefore, we believe that sEVs from NSCs may also promote functional recovery after traumatic SCI.

Studies have shown that autophagy is critical for protection against SCI^29,30^, possibly owing to the re-routing of metabolic substrates for repair. There is dynamic feedback between autophagy and cellular energy balance^31^. Autophagy has an important role not only in maintaining the supply of nutrients required for cell survival, but also as a regulator of cytoplasmic quality control by eliminating long-lived or unfolded proteins and impaired organelles^32,33^. In addition, autophagy induced by human umbilical cord mesenchymal stem cell-derived sEVs can effectively alleviate the nephrotoxicity of cisplatin^34^. These studies suggest that activation of autophagy can reduce tissue damage. However, it is unclear whether NSC-sEVs can activate autophagy.

Here, we show for the first time that NSC-sEVs have the potential to reduce neuronal apoptosis, inhibit neuroinflammation, and promote functional recovery in SCI model rats by activating autophagy.

## Material and methods

### Isolation and purification of NSC-sEVs

NSCs were obtained from 13.5-day fetal mouse spinal cord and cultured in growth medium with 2% N_2_ (Gibco, Grand Island, NY, USA), 1% B27 (R&D Systems, Minneapolis, MN, USA), bFGF 20 ng/ml (R&D Systems), and EGF 20 ng/ml (R&D Systems). The growth medium was collected and centrifuged at 300 g for 10 min, followed by centrifugation at 2000 × *g* for 10 min at 4 °C. After centrifugation, the medium supernatant was sterilized by filtration through a 0.22 μm filter to remove cellular debris. The upper compartment of the supernatant was then transferred to an Amicon Ultra-15 centrifugal filter (Millipore, Burlington, MA, USA) and centrifuge at 4000 × g at 4 °C until the volume of the upper chamber was reduced to ~ 200 μL. The ultrafiltrate was washed twice with phosphate-buffered saline (PBS) and ultrafiltered again to 200 μL. For sEV purification, the medium was loaded on a 30% sucrose/D2O pad in a sterile Ultra-ClearTM tube (Beckman Coulter, Asphalt, CA, USA) and centrifuged at 4 °C for 60 min at 100,000 × g using an optima L-100 XP Ultracentrifuge (Beckman Coulter). Partially purified NSC-sEVs were recovered using an 18 g needle, diluted in PBS, and centrifuged at 4 °C/4000 × g through the filter unit until the final volume reached 200 μL. The solution was stored at −80 °C or used immediately for experiments. The NSC-sEV protein content was determined using a bicinchoninic acid assay (BCA; Thermo Fisher Scientific, Waltham, MA) by measuring absorbance at 562 nm.

### Characterization of NSC-sEVs

To analyze the morphological characteristics of sEVs, a three-dimensional map of particle size, solid shape, and relative intensity was constructed using Nanosizer^TM^ (Malvern Instruments, Malvern, UK). The morphology of the obtained sEVs was also observed directly by transmission electron microscopy (TEM; Tecnai 12; Philips, Best, The Netherlands). Western blotting was used to detect the specific sEV surface markers CD9, CD63, and CD81.

### NSC-sEVs uptake

For sEVs fluorescent labeling, 4 mg/mL DiI solution (Molecular Probes, Eugene, OR, USA) was added to PBS (1:200) and incubated according to the manufacturer’s instructions. Excess dye from labeled sEVs was removed by ultracentrifugation at 100,000× g for 1 h at 4 °C. Isolated sEVs were washed three times by resuspending the pellet in PBS. The final pellet was resuspended in PBS. These DiI-labeled sEVs (DiI-sEVs) were co-cultured with neuronal cells or microglia for 24 h in vitro, and then the cells were washed with PBS and fixed in 4% paraformaldehyde. The uptake of DiI-sEVs was then observed by laser confocal microscopy. DiI-sEVs were also intravenously injected into the SCI site of model rats (described below) through the tail vein. After 2 h, the rats were anesthetized and the injured spinal cord removed for preparation of frozen tissue sections. Sections were stained with 4′,6-diamidino-2-phenylindole (DAPI) and observed under a fluorescence microscope.

### Primary spinal neuron culture

Embryonic (E16–E18) Sprague–Dawley (SD) rats were immersed in 75% ethanol, and the skin and cartilage were cut open along the back to dissect out the spinal cord. Spinal cords were placed in precooled Dulbecco’s modified Eagles medium/Nutrient Mixture F-12 (DMEM/F-12; Thermo Fisher Scientific, USA), rinsed, cut, and transferred to a centrifuge tube. Neurons were dissociated by digestion with 0.25% trypsin (Thermo Fisher Scientific) and 0.05% deoxyribonuclease I (Sigma-Aldrich, St. Louis, MO, USA) in a 37 °C incubator for 20 min. After the reaction was stopped by addition of horse serum (Sigma-Aldrich), cells were collected by centrifugation at 1000 rpm for 5 min at 4 °C, followed by resuspension in DMEM/F-12 containing 10% horse serum, penicillin (100 IU/mL), streptomycin (100 mg/mL; Thermo Fisher Scientific), and glutamine (0.5 mm; Thermo Fisher Scientific). After counting, cells were seeded on poly-d-lysine-coated plates (Corning Inc, Corning, NY, USA). For immunofluorescence staining, neurons were seeded in 24-well culture plates at 5 × 10^4^ cells/mL. For western blot assays, neurons were seeded at 1 × 10^6^ cells/mL in six-well culture plates. Culture plates were incubated for 4 h at 37 °C under a 5% CO_2_ atmosphere to allow adherence. The seed plate medium was then replaced with serum-free 96% Neurobasal medium containing B27 (2%, w/v; Thermo Fisher Scientific), glutamine (0.5 mm; Thermo Fisher Scientific), penicillin (100 IU/mL), and streptomycin (100 mg/mL). Half of the medium was changed every 2 days and cell growth was observed under an inverted microscope. Cells were cultured for 7 days before use in experiments. The purity of the neuronal cultures was assessed on day 7 by immunostaining with antibodies against microtubule associated protein-2 (MAP2; 1:500, rabbit IgG; Abcam, USA) and NeuN (1:800, mouse IgG; Abcam).

### Nitric oxide assay

Primary microglia were harvested from post-natal day 3 rat pups as described previously^35^. Microglia (2 × 10^5^ cells/mL) were plated on 96-well plates, pretreated with NSC-sEVs (100 μg/mL) for 1 h or left untreated (control), and then stimulated with 5 ng/mL lipopolysaccharide (LPS; Sigma-Aldrich). Supernatants were collected and the production of NO was measured every 24 h for 4 days using Griess reagent (Promega, Madison, WI, USA). In brief, the supernatant was mixed with an equal amount of Griess reagent and incubated for 10 min at room temperature. The absorbance was measured spectrophotometrically at 540 nm, and the NO concentration was extrapolated from the standard curve of sodium nitrite (NaNO_2_).

### In vitro cell apoptosis measurements by TUNEL staining or annexin V/FITC/PI double staining and flow cytometry

Exposure to glutamate (Glu; 100 μm) was used as an in vitro model of SCI-induced cell death. Cultured primary spinal neurons with or without NSC-sEVs (100 μg/mL) pretreatment for 24 h were subjected to Glu treatment and then terminal deoxynucleotidyl transferase dUTP nick end labeling (TUNEL) staining (Roche, Basel, Switzerland) at 37 °C for 30 min in the dark according to the manufacturer’s instructions. The cells were counterstained for 5 min with DAPI (Beyotime Biotechnology, China) and observed by a fluorescence microscope (AXIO Vert.A1 & Imager A2; Carl Zeiss Microscopy GmbH, Jena, Germany). Apoptotic cells and total cells were counted in randomly selected fields of view to calculate the proportion of TUNEL-positive (apoptotic) cells.

Apoptosis rate was also examined by flow cytometry. After the indicated treatment, cells were harvested by centrifugation at 1500 rpm for 5 min, and washed twice with PBS. The harvested cells were resuspended in fluorescein isothiocyanate (FITC)-labeled Annexin V (5 μL; BD Biosciences) and PI (5 μL; BD Biosciences) under darkness for 5 mins and washed three times with PBS. Cell apoptosis rate was then estimated by flow cytometry (FACSCalibur; BD Biosciences).

### Western blot analysis

Total protein was extracted from cells and tissues, and the protein concentration measured using a BCA assay as described. Proteins were separated by sodium dodecyl sulfate polyacrylamide gel electrophoresis and transferred onto polyvinylidene difluoride membranes. Membranes were blocked with 5% bovine serum albumin for 1 h at room temperature and incubated with antibodies against cleaved caspase-3 (1:1000, rabbit IgG; Cell Signal Technology, Danvers, MA, USA), Caspase-3 (1:1000, rabbit IgG; Cell Signal Technology), Bcl-2 (1:1000, rabbit IgG; Abcam, USA), Bax (1:1000, rabbit IgG; Abcam, USA), beclin-1 (1:1000, rabbit IgG; Abcam, USA), LC3B (1:1000, mouse IgG1; Abcam, USA), P62 (1:1000, mouse IgG1; Abcam, USA), GAPDH (as a gel-loading control, 1:1000, rabbit IgG; Abcam, USA), TNF-α (1:1000, mouse IgG1; Abcam, USA), IL-1β (1:1000, mouse IgG; Abcam, USA), and/or IL-6 (1:1000, mouse IgG; Abcam, USA). Membranes were then incubated with horseradish peroxidase-conjugated anti-rabbit IgG and anti-mouse IgG antibodies (1:2000, Thermo Fisher Scientific, USA) for 120 min, followed by visualization of the immunolabeled bands using an enhanced chemiluminescence reagent (Thermo Fisher Scientific, USA). Protein expression levels were determined by densitometry using ImageJ (NIH, Bethesda, MD).

### Immunofluorescence staining of cultured neurons

Cells treated as described were fixed with precooled paraformaldehyde (4%, w/v) for 20 min, then permeabilized with 0.2% Triton X-100 for 20 min, blocked with 10% normal goat serum, and finally incubated overnight at 4 °C with the following primary antibodies: anti-MAP2 (1:500, rabbit IgG; Abcam, USA), anti-NeuN (1:800, mouse IgG; Abcam, USA), anti-Nestin (1:500, mouse IgG1; BD Biosciences, USA), anti-SOX2 (1:500, rabbit IgG; Abcam, USA), and/or anti-cleaved caspase-3 (1:1000, rabbit IgG; Cell Signal Technology, USA). The following day, the cells were treated with secondary antibody at room temperature for 1 h and the nuclei were counterstained for 10 min with DAPI. Immunoreactivity was visualized using a fluorescence microscope (AXIO Vert.A1&Imager A2, Carl Zeiss Microscopy GmbH, Germany).

### Rat model of SCI and experimental groups

Healthy adult male Sprague–Dawley rats (weighing 180−220 g) were purchased from the Animal Center of Nanjing Medical University (Nanjing, Jiangsu, China). Rats were housed in a specific pathogen-free laboratory animal center under a controlled temperature (23 ± 0.5 °C) and a 12:12 h light:dark cycle. The study was approved by the Ethics Committee of Nanjing Medical University. All procedures were conducted in accordance with the guidelines of the National Institutes of Health Laboratory Animal Care and Use Guidelines.

A rat model of SCI was established using a modification of the Allen method. As described in previous studies^36^, after anesthesia, rat skin preparation, and precise positioning, laminectomy was conducted to expose the T10 spinal cord. The exposed back surface of the spinal cord was subjected to weight impact contusion using a 10-g rod (2.5 mm in diameter; C4p01–001; RWD Life Science Corp, Shenzhen, China) dropped from a height of 12.5 mm. After the impact, successful SCI was verified by body trembling, swaying of the tail, and a fluttering retraction of the hind limbs and body. In successful models, the hind limbs then displayed flaccid paralysis, and edema could be observed on the dural surface. After injury, the spinal cord was washed with saline, the incision sutured, and antibiotic treatment given for 3 consecutive days. Rats also received artificial bladder drainage three times a day until bladder function was restored.

Rats fulfilling these SCI model criteria were randomly divided into two groups, an SCI-only group and a NSC-sEVs pretreatment group (*n* = 8/group). Sham operated rats served as the control (*n* = 8/group). The SCI and NSC-sEVs group rats were administered physiological saline (PBS, 200 μL) and NSC-sEVs (200 μg of total protein in 200 μL of PBS), respectively, by tail vein injection immediately after SCI.

### Assessment of locomotor capacity

The well-established Basso−Beattie−Bresnahan (BBB) scoring method was used to evaluate motor function. This method comprehensively assesses rat motor function by measuring hind-limb joint activities and range of motion, load-bearing capacity, coordination of the front and rear limbs, and movement of the front and rear paws and tail. Prior to testing, the bladder was emptied. Rats were then placed on the ground, and the hind-limb activity was recorded. The scores were measured before surgery and then on the 1st, 3rd, 7th, 14th, 21st, and 28th day post-surgery. A score out of 21 points was given, with 0 points indicating full hind-limb paralysis. Assessments began at a fixed time in the morning of each testing day and were performed independently by two trained experimenters blind to treatment history.

### Footprint analysis

Gait and motor coordination were evaluated 28 days post surgery. The front and rear paws were coated with dyes of different colors. A rat was then placed on a piece of absorbent paper surrounded by a cage to encourage the animal to walk in a straight line. The footprint pattern was then digitized and a representative picture used to assess coordination.

### Nissl staining of unfixed spinal cord sections

The cytosolic Nissl substance in spinal cord sections was stained with cresyl violet on the 28th day after surgery. In brief, sections were washed with distilled water and stained for 10 mins in a cresyl violet solution. After rinsing with distilled water, the sections were differentiated with 95% ethanol, washed with xylene, and fixed with neutral balsam. Regions of traumatic injury were identified by serious tissue destruction or loss of staining. Five Nissl-stained sections were randomly selected to estimate the average number of anterior horn motoneurons remaining and the proportional lesion size.

### Magnetic resonance imaging (MRI)

Three animals were randomly selected from each group for MRI on the first day post surgery. Rats were anesthetized with halothane (3−4% induction, 1.5−2% maintenance) in oxygen (0.4 L/min) and nitrogen (0.6 L/min). An anesthetized rat was placed prone on the fixation system and examined using a small animal MRI system (Bruker BioSpec7T/20 USR; Bruker AXS GmbH, Karlsruhe, Germany). The sequence protocol was executed using the following parameters: T2-weighted; 256 × 256 matrix; slice thickness, 1 mm; intersection gap, 1 mm; echo time/repetition time: 27/3000 ms; rapid acquisition with relaxation enhancement factor, 16; flip angle, 90 degrees. T2-weighted images were acquired in sagittal and axial planes with ParaVision (version 6.0.1, Bruker BioSpec; Bruker AXS GmbH).

### Preparation of spinal cord slices

Rats were anesthetized with a lethal dose of chloral hydrate. The skin of the chest and abdomen was then cut, the xiphoid lifted, the thoracic cavity opened along the ribs, and the sternum clamped backwards with a hemostat to fully expose the chest cavity. An empty needle was inserted into the heart and fixed in place, and a small gap in the right atrial appendage was cut. Cold saline was infused through the heart until the viscera of the rat became colorless and liquid overflowed from the right atrial appendage, indicating that the blood had been replaced by saline. Paraformaldehyde (PFA; 4% w/v) was then infused until the limbs and trunk of the rat became stiff. The spine was then removed, the lamina opened, and the spinal cord carefully dissected while maintaining tissue integrity. The injury site was marked as the center, and a segment of spinal cord including 1 cm of tissue on either side of the injury site was collected. The tissue was fixed in PFA (4% w/v) for 24 h at 4 °C, and transferred to PBS with 20% sucrose (w/v), followed by 30% sucrose–PBS until the tissue had sunk to the bottom of the container. Tissue was then embedded in optimal cutting temperature compound, and sliced along the longitudinal axis at a thickness of 18 µm using a freezing cytotome. Sections were then collected onto a poly-d-lysine-coated anti-offset slide. All slides were stored at −80 °C until further analysis.

### TUNEL staining of spinal cord slices

Spinal cord sections prepared as described were fixed, blocked, and incubated with anti-NeuN (1:800, rabbit IgG; Abcam, USA) overnight at 4 °C. The sections were washed with PBS and incubated with Alexa Fluor 488-conjugated goat anti-rabbit IgG (1:200, Jackson ImmunoResearch, West Grove, PA, USA) for 2 h, and then reacted with the TUNEL reaction mixture (Promega) at 37 °C for 1 h. The nuclei were counterstained with DAPI. The proportion of TUNEL-positive neurons in each group of animals was counted under a fluorescence microscope.

### Spinal tissue immunofluorescence staining

Spinal cord sections prepared as described were permeabilized in Triton X-100 PBS solution (0.3% w/v) for 30 min, blocked using native goat serum-PBS solution (10%, v/v), and stained overnight at 4 °C with primary antibodies against the following proteins: CD68 (1:200, mouse IgG1; EMD Millipore Corp), GFAP (1:1000, rabbit IgG1; Abcam, Cambridge, UK), LC3B (1:200, mouse IgG; Abcam, USA), beclin-1(1:200, rabbit IgG; Abcam, USA), and/or NeuN (1:500, rabbit IgG; Abcam, USA and 1:500, mouse IgG; Abcam, USA), and/or cleaved caspase-3 (1:200, rabbit IgG; Cell Signal Technology, USA). Sections were washed three times in PBS, and incubated with Cy3- or FITC-conjugated secondary antibodies (1:200, Jackson ImmunoResearch, USA) for 2 h at room temperature. Nuclei were then counterstained using DAPI, and fluorescent images were acquired. For each slide, the SCI lesion area was identified as the region lacking staining. All images were acquired using the same exposure time and conditions for comparison among animals and groups.

### Double-labeled adenovirus mRFP-GFP-LC3 transfection and autophagy detection

Primary spinal cord neurons prepared as described were seeded on confocal dishes for 4 days, and then transfected with mRFP-GFP-LC3 lentivirus (Han Heng Biology, China) according to the manufacturer’s protocol. Cells were divided into three groups: control, Glu, and NSC-sEVs + Glu. Following treatment, cells were washed with PBS, fixed in 4% paraformaldehyde, and observed by laser confocal microscopy (Zeiss, Oberkochen, Germany, LSM 510). The number of yellow spots representing autophagic bodies and red spots representing autophagic lysosomes were counted.

### Cyto-ID kit® autophagy detection kit for detection of autophagy

Primary spinal neurons treated as indicated were washed twice with 100 μL 15% FBS lx Assay Buffer then incubated in Cyto-ID dye (Enzo Life Sciences, Farmingdale, NY, USA) at 37 °C for 30 min in the dark. The staining solution was discarded and cells were washed with 100 μL 15% FBS in lx Assay Buffer to remove unbound dye. Cells were fixed in 4% paraformaldehyde and examined for green autophagosome labeling by fluorescence microscopy (AXIO Vert.A1&Imager A2, Carl Zeiss Microscopy GmbH, Germany).

### TEM assessment autophagy

After treatment, adherent neurons were deplated with trypsin and centrifuged. The cell pellet was fixed with a precooled 2% glutaraldehyde solution at 4 °C for 2 h. The cells were stained with 2% uranyl acetate solution for 2 h and then dehydrated in 50%, 70%, 90%, and 100% acetone. The cells were embedded and ultrathin sections prepared for observation under an electron microscope (FEI Tecnai, Hillsboro, OR, USA).

### Statistical analysis

**Fig. 1:**
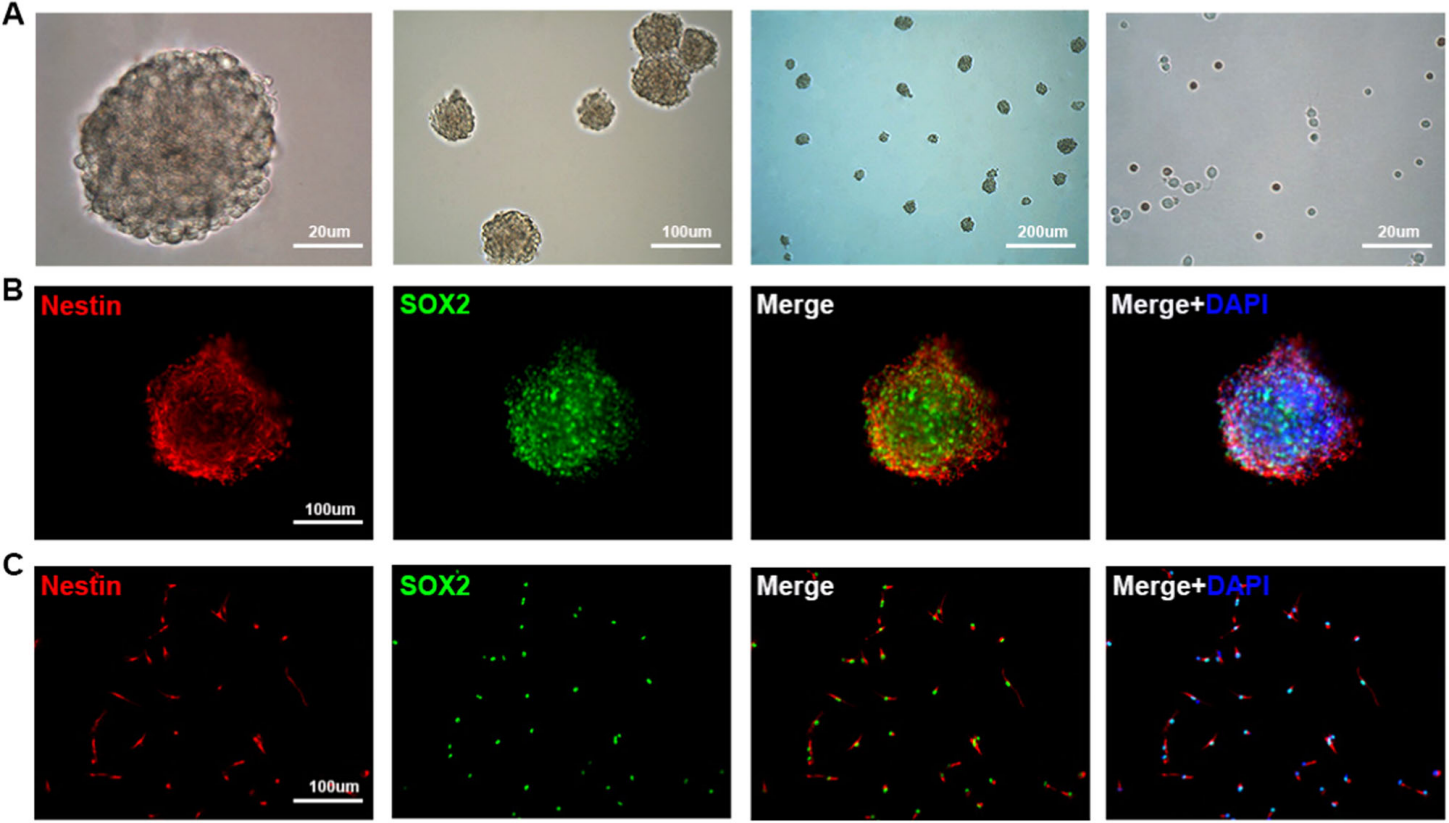
Characteristics of neural stem cells (NSCs) in culture. **a** Neurospheres and free single cells showing typical morphological features. **b** Immunofluorescence staining of adherent neurospheres showing expression of the NSC markers nestin (red) and SOX2 (green). **c** Immunofluorescence staining of individual NSCs showing nestin (red) and SOX2 (green) expression. Cell nuclei were counterstained with DAPI (blue). DAPI, 4’,6-diamidino-2-phenylindole

**Fig. 2:**
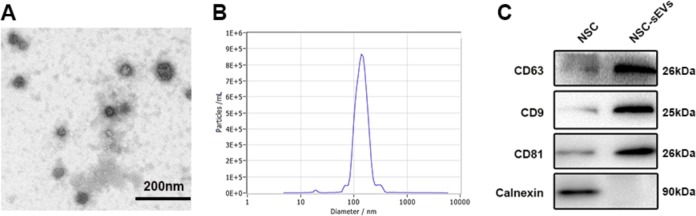
Characterization of NSC-derived small extracellular vesicles (NSC-sEVs). **a** Small extracellular vesicle morphology revealed by transmission electron microscopy (TEM). **b** Particle size distribution measured by dynamic light scattering (DLS). **c** Western blot analysis of specific small extracellular vesicle surface markers

**Fig. 3:**
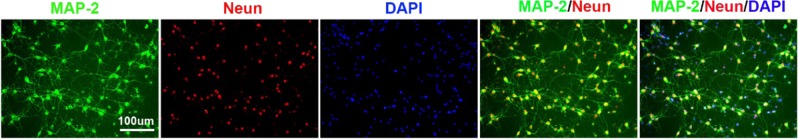
Immunocytochemical identification of primary rat spinal cord neurons. Neuronal dendrites and axons were identified by anti-MAP2 (green) and somata by NeuN (red) immunostaining. The nuclei of all cells were identified by DAPI (blue)

**Fig. 4:**
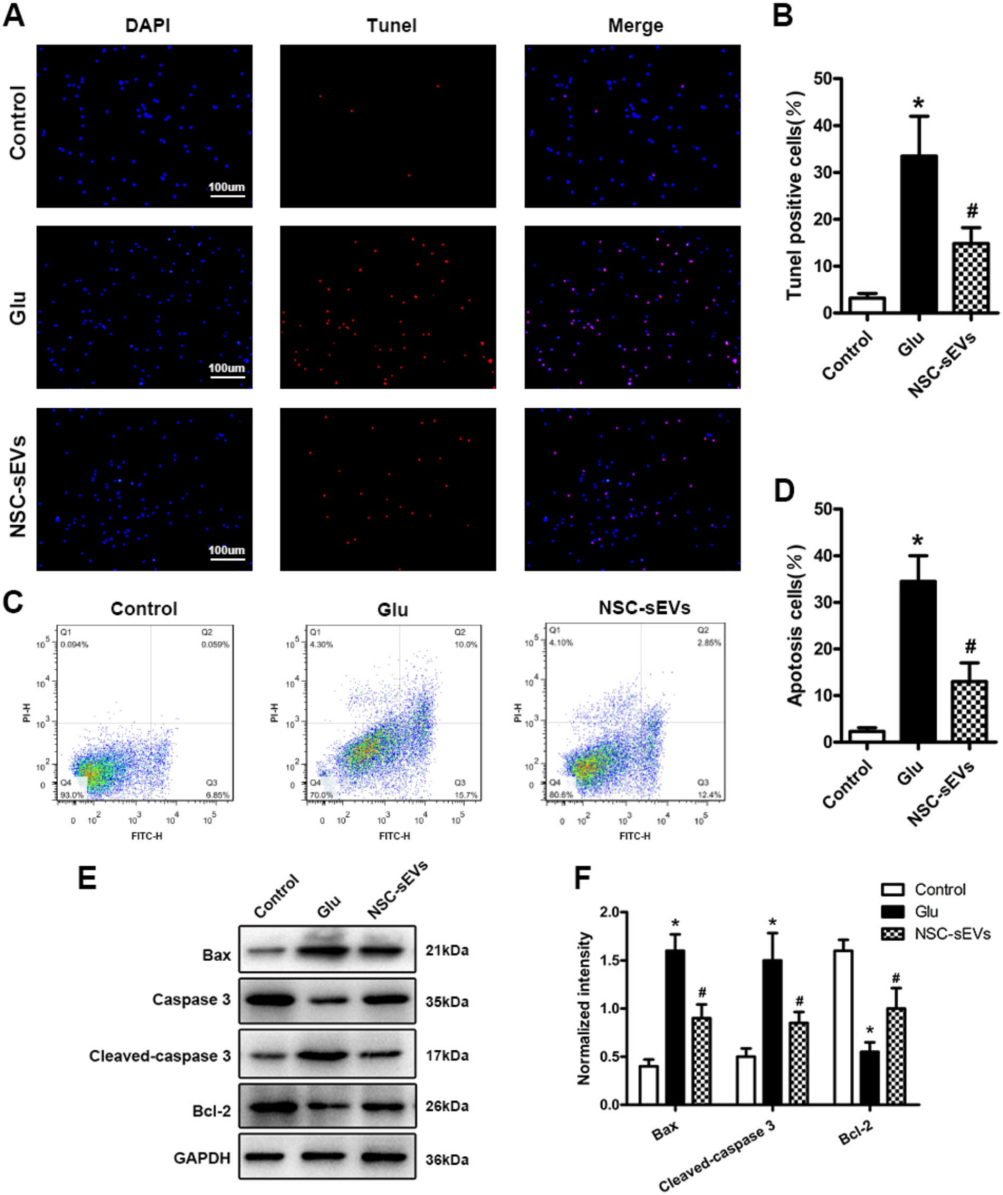
NSC-sEV pretreatment attenuates glutamate-induced neuronal apoptosis in vitro. **a** TUNEL staining (red) for detection of apoptosis in primary rat spinal cord neurons. Cell nuclei were counterstained with DAPI (blue). **b** Quantitative estimation of the proportion of apoptotic cells in each experimental group: control (untreated), glutamate alone (Glu, 100 μm, 30 min), and NSC-sEV pretreatment (100 μg/mL, 24 h) followed by glutamate as indicated (NSC-sEVs + Glu). Pretreatment with NSC-sEVs substantially reduced Glu-induced apoptosis. **c** Annexin V/FITC/PI double staining flow cytometry was also used to detect neuronal apoptosis induced by Glu with or without NSC-sEV pretreatment. **d** Quantitative results of flow cytometry confirming that NSC-sEV pretreatment reduced Glu-induced apoptosis of primary spinal cord neurons. **e** Western blot analysis of neuronal apoptosis-related proteins. **f** Relative expression levels of apoptosis-related proteins normalized to GAPDH. Pretreatment with NSC-sEVs upregulated anti-apoptotic Bcl-2 and downregulated proapoptotic Bax and cleaved caspase-3. *p < 0.05 compared with the Control group, ^#^*p* < 0.05 compared with the Glu alone group. NSC-sEVs, neural stem cell-derived small extracellular vesicles; Bax, Bcl-2-associated X protein; Bcl-2, B-cell lymphoma 2; FITC, fluorescein isothiocyanate; PI, propidium iodide; GAPDH, glyceraldehyde 3-phosphate dehydrogenase; Glu, glutamate; TUNEL, terminal deoxynucleotidyl transferase-mediated dUTP nick end labeling

**Fig. 5:**
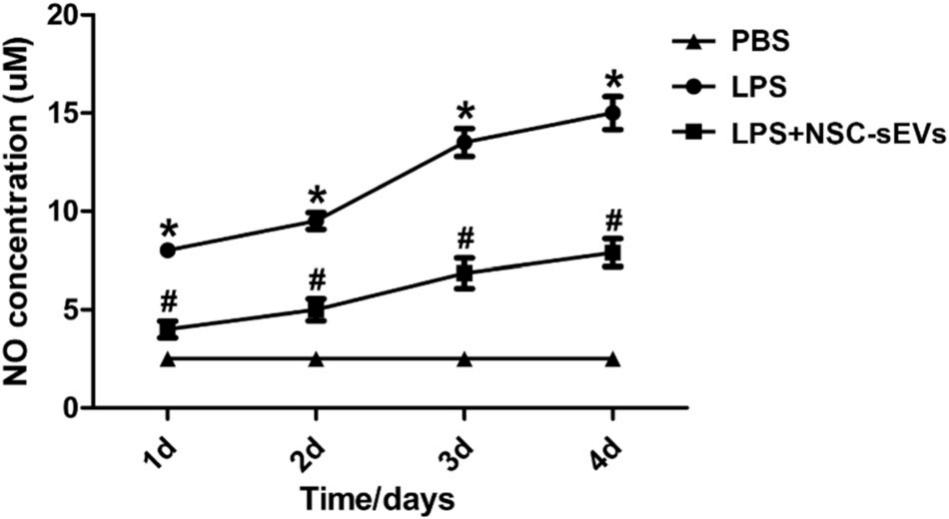
Effect of NSC-sEV pretreatment on microglia function. Lipopolysaccharide (LPS) markedly increased the production of nitric oxide (NO) by microglia in vitro, a response significantly attenuated by NSC-sEV pretreatment. *p < 0.05 compared with the PBS group, ^#^*p* < 0.05 compared with the LPS group

**Fig. 6:**
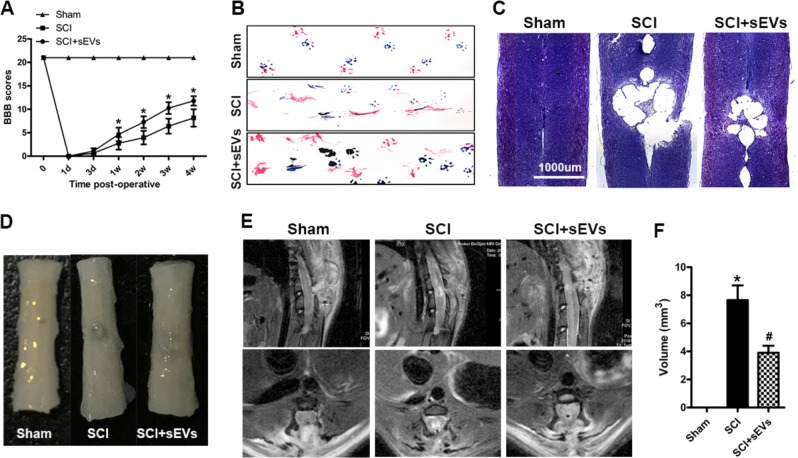
NSC-sEV pretreatment promotes functional recovery after SCI and reduces the area of damage. **a** Basso, Beattie, and Bresnahan (BBB) limb function scores at different times after spinal cord contusion. **b** Representative footprints of an animal walking 28 days after SCI. Blue: front paw print; red: hindpaw print. **c** Representative Nissl-stained sagittal section of spinal cord. **d** Gross morphology of spinal sections. **e** Representative sagittal and coronal MRI images. **f** Quantitative analysis of lesion volume in sham, SCI, and SCI + NSC-sEVs treatment groups. *p < 0.05 compared with the Sham group, ^#^*p* < 0.05 compared with the SCI group

**Fig. 7:**
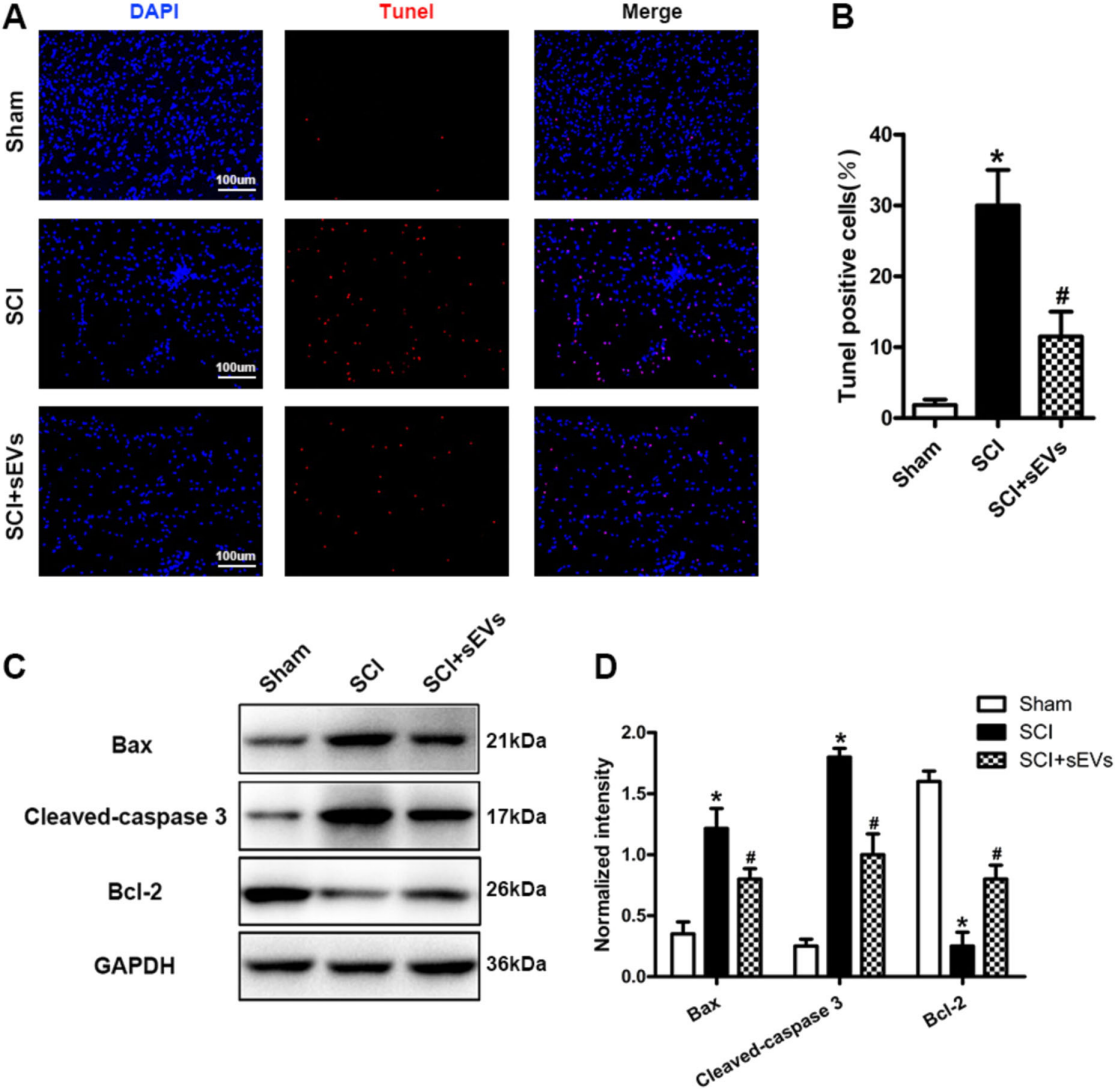
NSC-sEV pretreatment protects spinal neurons from apoptosis following SCI. **a** TUNEL staining was used to detect neuronal apoptosis in the spinal cord following injury. **b** Quantification of TUNEL-positive neurons in each experimental group. The proportion of TUNEL-positive neurons was significantly lower in the SCI + NSC-sEVs group than the SCI group. **c** Western blot analysis of apoptosis-related proteins after SCI. **d** Semi-quantitative analysis (normalized to GAPDH) showing that the increase in proapoptotic proteins Bax and cleaved caspase-3 following SCI was reversed by NSC-sEVs pretreatment, whereras expression of the anti-apoptotic Bcl-2 was higher in the SCI + NSC-sEVs group than the SCI group. *p < 0.05 compared with the Sham group, ^#^*p* < 0.05 compared with the SCI group. NSC-sEVs, neural stem cell-derived small extracellular vesicles; Bax, Bcl-2-associated X protein; Bcl-2, B-cell lymphoma 2; DAPI, 4’,6-diamidino-2-phenylindole; GAPDH, glyceraldehyde 3-phosphate dehydrogenase; TUNEL, terminal deoxynucleotidyl transferase-mediated dUTP nick end labeling; SCI, spinal cord injury

**Fig. 8:**
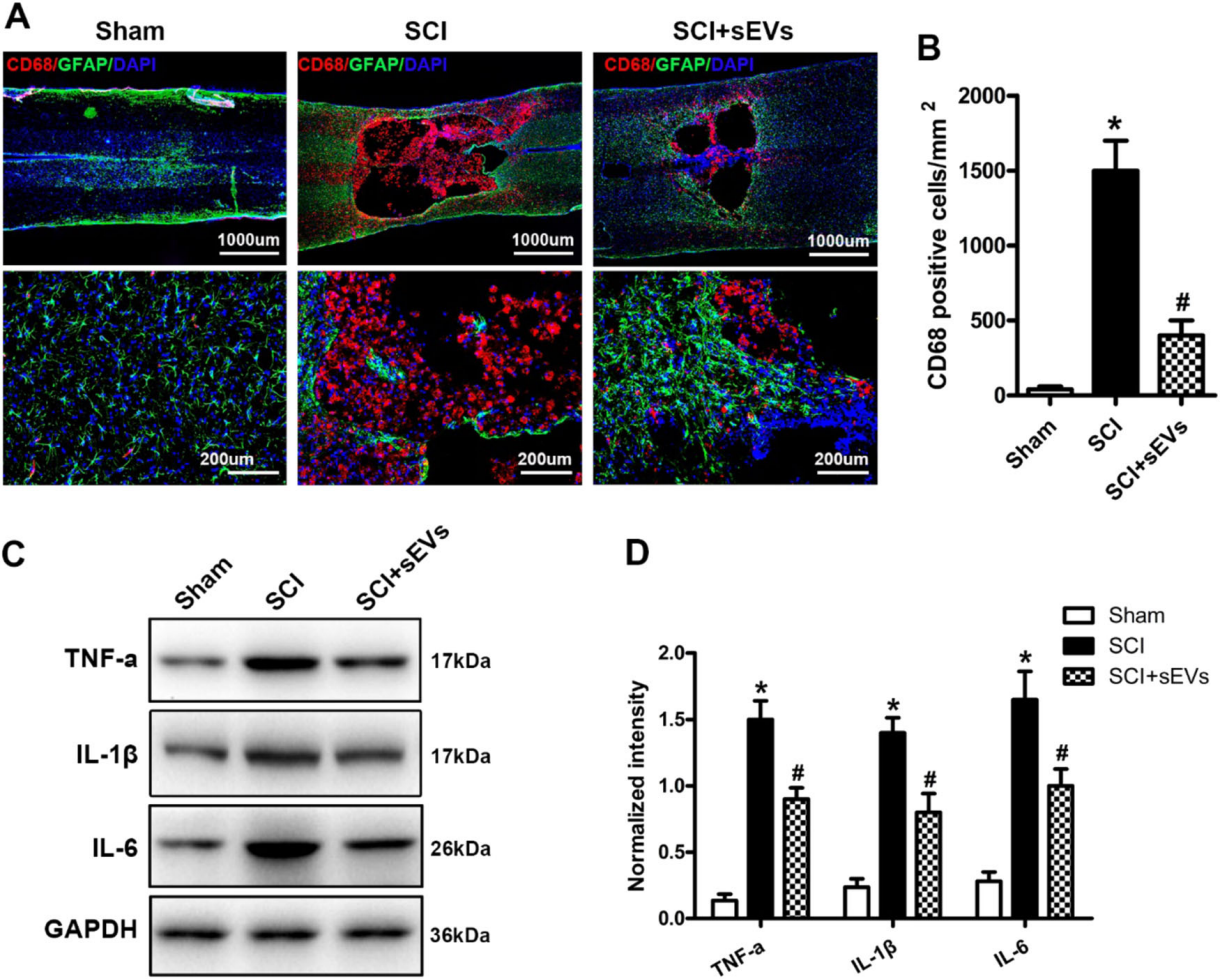
NSC-sEV pretreatment inhibits microglial activation and reduces neuroinflammation following SCI. **a** Representative images of CD68 (red) and GFAP (green) immunohistochemical staining on day 3 after injury in the SCI and SCI + NSC-sEVs groups. All cell nuclei were counterstained with DAPI (blue). **b** Numbers of CD68-positive (activated) microglia in the injury area. **c** Western blot detection of inflammation-related proteins. **d** Semi-quantitative analysis of inflammation-related protein levels. NSC-sEVs substantially reduced the expression levels of pro-inflammatory cytokines TNF-α, IL-1β, and IL-6 following SCI. *p < 0.05 compared with the Sham group, ^#^*p* < 0.05 compared with the SCI group. NSC-sEVs, neural stem cell-derived small extracellular vesicles; SCI, spinal cord injury; DAPI, 4’,6-diamidino-2-phenylindole; GAPDH, glyceraldehyde 3-phosphate dehydrogenase; GFAP, glial fibrillary acidic protein; IL, interleukin; TNF-α, tumor necrosis factor alpha; IL-1β, interleukin-1β; IL-6, interleukin-6

**Fig. 9:**
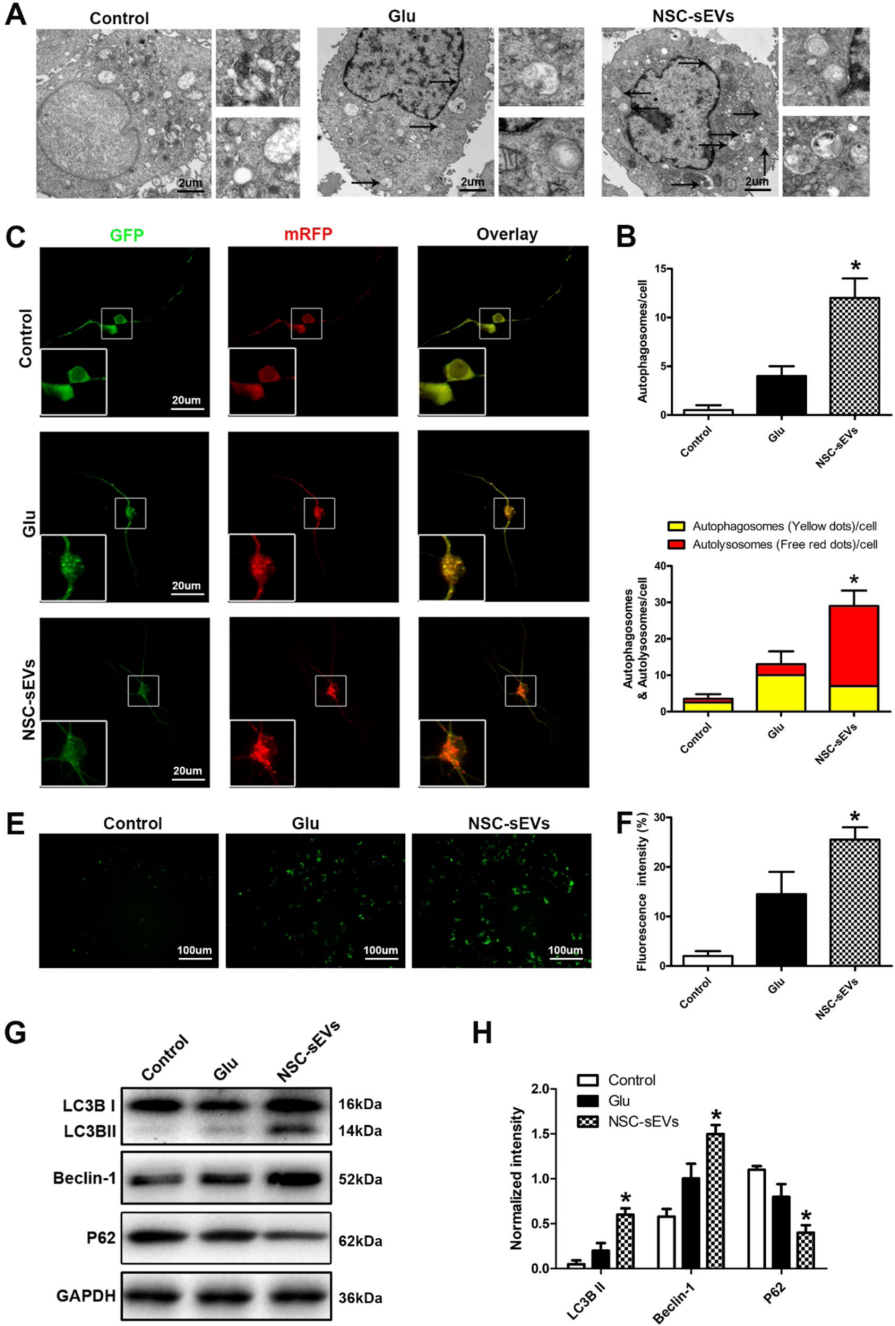
NSC-sEV pretreatment induces autophagy in primary spinal cord neurons. **a**, **b** Transmission electron micrographs of autophagosomes in NSC-sEV-pretreated spinal neurons. **c**, **d** Autophagic flux of mRFP-GFP-LC3-transfected spinal neurons revealed by laser confocal microscopy. Autophagosomes are labeled by red and green fluorescence (yellow spots), whereas autophagic lysosomes are labeled by red fluorescence (red spots). The NSC-sEVs + Glu group demonstrated a larger number of yellow and red spots than the Glu-only group. **e**, **f** CYTO-ID detection of autophagosome formation. **g** Western blot detection of neuronal autophagy markers LC3B and Beclin-1. **h** Semi-quantitative analysis showing enhanced expression of autophagy-related proteins in NSC-sEV-pretreated primary spinal neurons compared with controls. *p < 0.05 compared with the Glu group. NSC-sEVs, neural stem cell-derived small extracellular vesicles

**Fig. 10:**
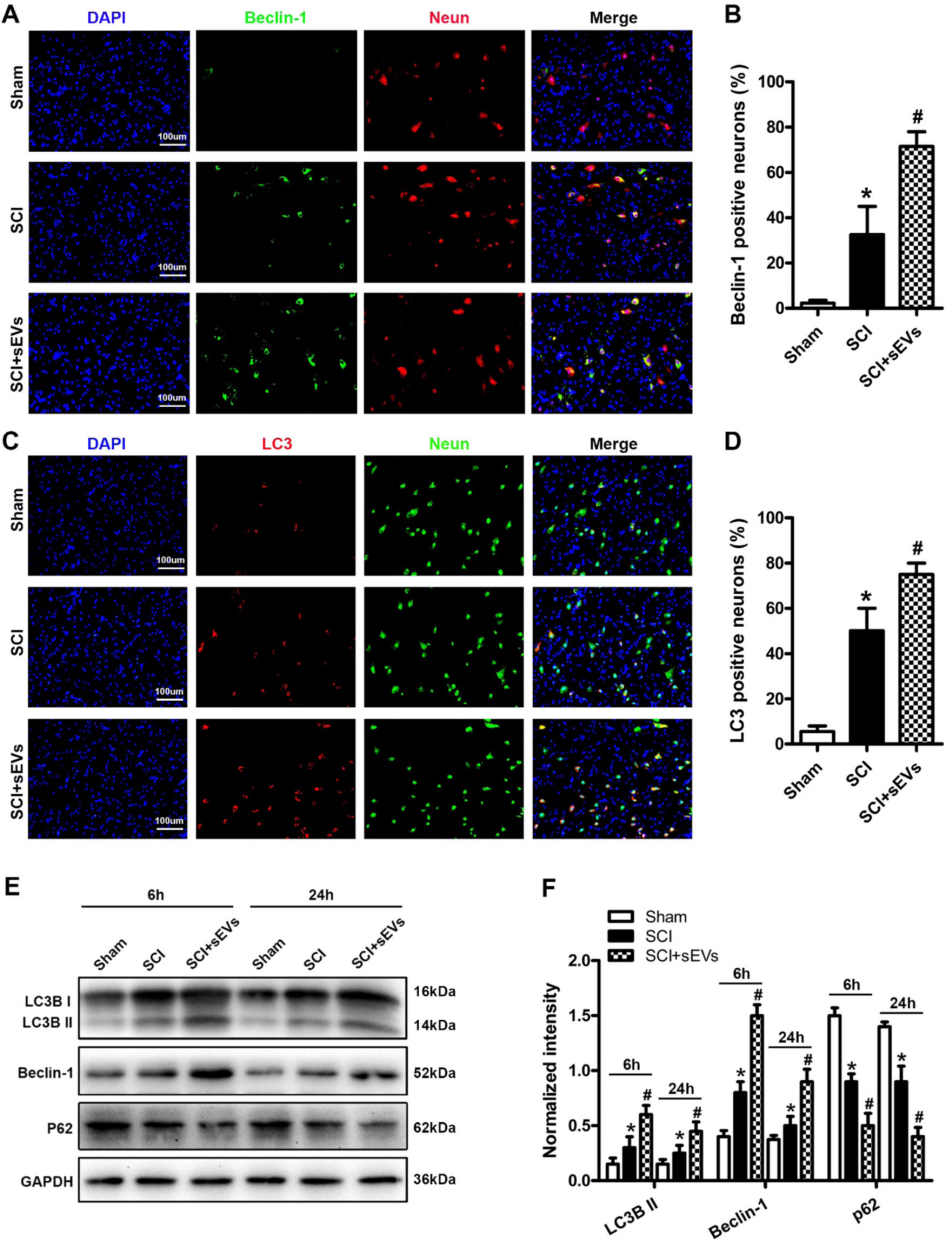
Immunofluorescence and western blot analysis of beclin-1 and LC3B expression in spinal cord following injury. **a**, **b** Numbers of beclin-1/NeuN/DAPI double positive neurons in spinal sections as determined by fluorescence microscopy. Beclin-1-positive (autophagic) neurons were significantly more numerous in the SCI + NSC-sEVs group than the SCI group. **c**, **d** Number of LC3B/NeuN/DAPI double-positive neurons. LC3B-positive neurons were also significantly more numerous in the SCI + NSC-sEVs group. **e** Western blot analysis of autophagy-related proteins at 6 and 24 h after SCI. **f**)Semi-quantitative analysis showing significantly greater expression levels of autophagy-related proteins in the SCI + NSC-sEVs group than the SCI group. Expression was normalized to GAPDH. *p < 0.05 compared with the Sham group, ^#^*p* < 0.05 compared with the SCI group. NSC-sEVs, neural stem cell-derived small extracellular vesicles; SCI, spinal cord injury; GAPDH, glyceraldehyde 3-phosphate dehydrogenase

**Fig. 11:**
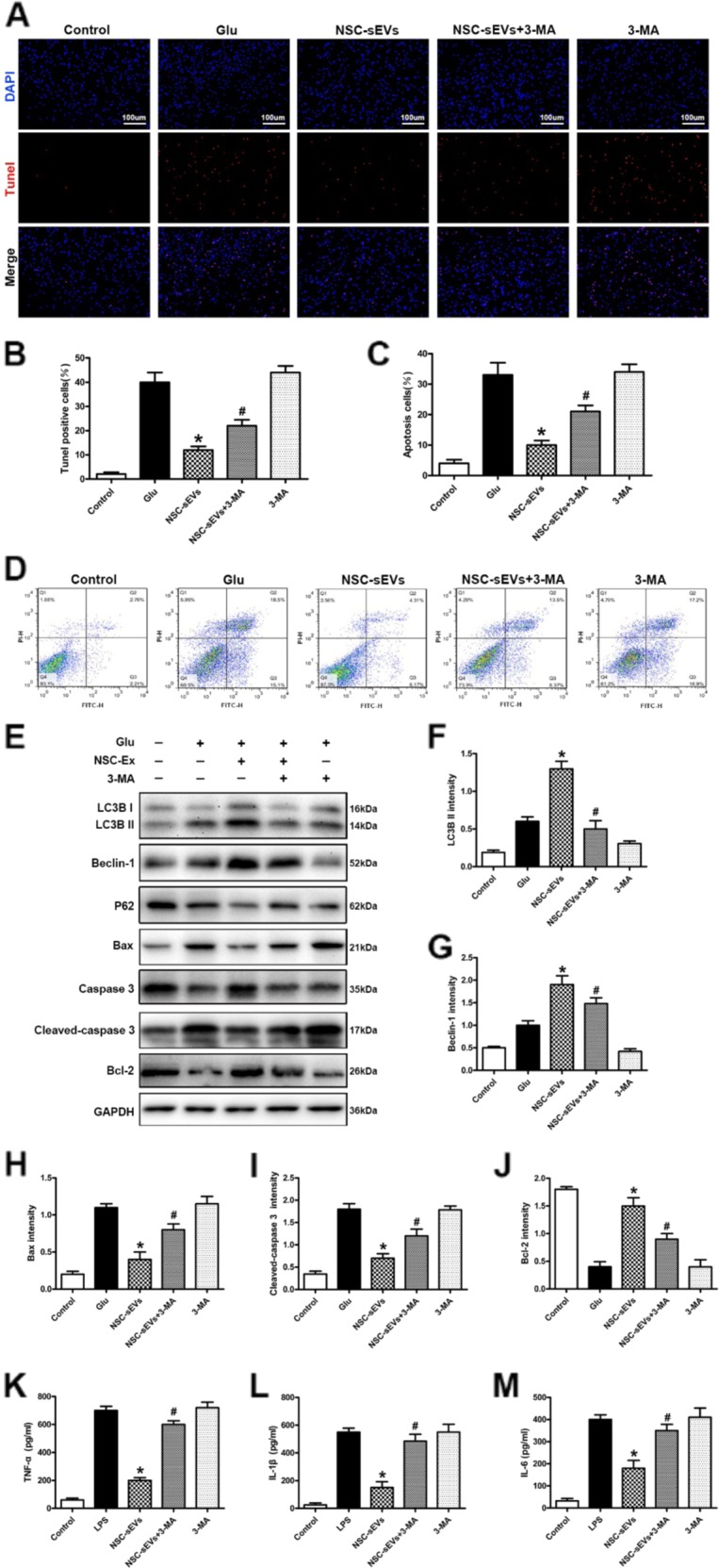
NSC-sEVs reduce apoptosis and secretion of inflammatory factors by activating autophagy. **a** TUNEL staining was used to detect neuronal apoptosis induced by Glu with or without NSC-sEVs and 3MA pretreatment. **b** TUNEL-positive neuron numbers. The reduction in TUNEL-positive neurons observed in the Glu + NSC-sEVs group compared with the Glu group was reversed by co-treatment with 3MA. **c**, **d** Neuronal apoptosis was detected by Annexin V/FITC/PI double staining and flow cytometry. Results were consistent with TUNEL staining. **e** Western blot was used to detect the expression of autophagy- and apoptosis-related proteins. 3MA pretreatment inhibited NSC-sEV-induced elevations in autophagy marker proteins (LC3B and beclin-1) and the anti-apoptotic protein Bcl-2, and reversed the NSC-sEV-induced decrease in proapoptotic Bax and cleaved caspase-3 expression. **f**–**j** Semi-quantification of autophagy and expression levels of apoptosis-related proteins. **k**–**m** ELISA analysis of TNF-α, IL-1β, and IL-6 expression by LPS-treated (activated) macrophages. The suppression of TNF-α, IL-1β, and IL-6 production by NSC-sEV pretreatment was reversed by 3MA pretreatment. *p < 0.05 compared with the Glu or LPS group, ^#^*p* < 0.05 compared with the NSC-sEVs group. NSC-sEVs, neural stem cell-derived small extracellular vesicles; TUNEL, terminal deoxynucleotidyl transferase-mediated dUTP nick end labeling; Glu, glutamate; 3MA, 3-methyladenine; FITC, fluorescein isothiocyanate; PI, propidium iodide; Bax, Bcl-2-associated X protein; Bcl-2, B-cell lymphoma 2; TNF-α, tumor necrosis factor alpha; IL-1β, interleukin-1β; IL-6, interleukin-6; LPS, lipopolysaccharide

Data and images were processed and analyzed using IBM SPSS Statistics v17.0. Data are expressed as mean ± standard deviation of at least three independent experiments. To analyze the changes in BBB score over time and differences between groups, we used a two-factor repeated measures analysis of variance with Bonferroni’s post hoc correction for multiple comparisons. Other data were analyzed using the Student’s *t* test. All tests were two-tailed and a *p* value < 0.05 was accepted as statistically significant for all tests (Figs. 1–11).

## Results

### Characterization of NSCs cultured in vitro

Stem cells obtained from E13.5 mouse spinal cord and cultured in growth medium proliferated into neurospheres of roughly uniform size with typical shape and refractive index (Fig. 1a). Both neurospheres and free single cells were immunopositive for the stem cell markers nestin (green fluorescence) and sex determining region Y -box 2 (Sox2) (red fluorescence) (Fig. 1b, c).

### Characterization of NSC-sEVs

Small extracellular vesicles were isolated from the NSC culture supernatant by a combination of centrifugation, ultrafiltration, and ultracentrifugation. The purified NSC nanoparticles were identified by transmission electron microscopy (TEM), dynamic light scattering (DLS) analysis, and western blotting. TEM revealed typical sEV structures, and DLS analysis revealed a particle size distribution between 20 and 150 nm, similar to previous reports (Fig. 2a, b). In addition, western blotting revealed the presence of the sEV surface markers CD63, CD9, and CD81 (Fig. 2c). Calnexin were not detected among sEV proteins. Collectively, these analyses confirmed successful isolation of sEVs from NSC cultures (Fig. 2c).

To investigate the feasibility of using NSC-sEVs for treatment of SCI, we examined cellular uptake of labeled sEVs (DiI-sEVs) both in vitro and in vivo. First, cultured neuronal cells and microglia were incubated with DiI-sEVs and uptake examined by fluorescence microscopy. After 24 h, DiI-sEVs were observed in the cytoplasm, confirming uptake (Supplementary Fig. S1a, b). In vivo experiments further demonstrated that DiI-sEVs can accumulate at sites of SCI and be taken up by nerve cells following injection (Supplementary Fig. S1c). Taken together, these data suggest that NSC-sEVs can be delivered to regions of SCI by systemic administration.

### Identification of primary neuronal cells cultured in vitro

The purity of primary spinal cord neuron cultures was confirmed by immunopositivity for MAP2, a marker of mature neurons^37^. Cells isolated from neonatal rat spinal cord also exhibited typical neuronal morphology with DAPI-stained somata and MAP2-positive axons and dendrites (Fig. 3).

### NSC-sEVs reduce neuronal apoptosis in vitro

High levels of extracellular Glu can lead to neuronal cell death (excitotoxicity), and Glu-induced toxicity is one of the most important pathogenic mechanisms for neuronal apoptosis and neurological dysfunction in SCI^38,39^. Therefore, we used TUNEL to study the protective effect of NSC-sEV pretreatment against Glu-induced apoptosis in primary neurons. Glutamate-treated cells (100 μm, 30 min, 37 °C) demonstrated a relatively high apoptotic ratio (TUNEL positive to total cells) compared with untreated controls that was significantly reduced by 24 h pretreatment with NSC-sEVs (100 μg/mL), indicating that NSC-sEVs have neuroprotective effects against Glu-induced excitotoxicity (Fig. 4a, b).

Next, we evaluated apoptosis by Annexin V-FITC/PI double staining and flow cytometry. As shown, exposure to Glu significantly increased apoptosis compared with untreated controls, and consistent with TUNEL-staining results, this response was significantly attenuated by pretreatment with NSC-sEVs (Fig. 4c, d).

Western blotting revealed that expression levels of the proapoptotic proteins Bax and cleaved caspase-3 were significantly downregulated, whereas expression of the anti-apoptotic protein Bcl-2 was elevated in spinal neurons pretreated with NSC-sEVs for 24 h prior to Glu treatment compared with spinal neurons treated with Glu only. In addition, immunofluorescence staining of cleaved caspase-3 further demonstrated that NSC-sEVs protect neurons from Glu-induced apoptosis in vitro (Supplementary Fig. S2).

### NSC-sEVs suppressed lipopolysaccharide-induced nitric oxide production by macrophages

Nitric oxide production by macrophages was markedly elevated by LPS (5 ng/mL) treatment compared with PBS-treated controls, and NSC-sEV pretreatment (100 μg/mL) significantly reduced this LPS-induced NO production at 24, 48, 72, and 96 h (Fig. 5). However, NO production remained significantly higher than the PBS-treated control group (Fig. 5).

### NSC-sEVs treatment improved functional recovery and reduced lesion volume following traumatic SCI

To test the neuroprotective efficacy of NSC-sEVs in vivo, we performed motor function assessments and MRI on SD rats with weight-induced spinal contusion at T10. Motor function in the SCI + NSC-sEVs group and the SCI-only group gradually improved over the first week following SCI as evidenced by BBB scores (Fig. 6a). However, BBB scores continued to increase in the NSC-sEVs group and were significantly higher than in the SCI group within 2−4 weeks after SCI (Fig. 6a). Coordination of forepaw−hindpaw movements decreased significantly immediately after SCI as determined by gait analysis, but animals treated with NSC-sEVs showed significantly faster gait recovery and improved motor coordination compared with SCI group animals (Fig. 6b). Motor performance of the sham-surgery group remained unchanged throughout the test period. Also, the lesion area was significantly smaller in the SCI + NSC-sEVs treatment group than the SCI-only group (Fig. 6c, d and f). Nissl staining revealed a significant loss of SCI tissue in the SCI group at 4 weeks post injury that was significantly reduced in the SCI + NSC-sEVs treatment group (Fig. 6c). Finally, MRI conducted in a randomly chosen subset of each group revealed significantly reduced lesion size in the SCI + NSC-sEVs treatment group compared with the SCI group (Fig. 6e).

### NSC-sEVs attenuated neuronal cell death in injured spinal cord

TUNEL staining was used to evaluate neuronal apoptosis in the area of SCI. On the first day after injury, the number of TUNEL-positive (apoptotic) cells in the SCI + NSC-sEVs group was significantly lower than in the SCI group (Fig. 7a, b). Further, western blot analysis of spinal lysates revealed higher expression levels of the apoptosis-related markers Bax and cleaved caspase-3 in the SCI group compared with the sham and SCI + NSC-sEVs groups, whereas the expression level of the anti-apoptotic protein Bcl-2 was significantly higher in the SCI + NSC-sEVs group than the SCI group, consistent with TUNEL staining and in vitro apoptosis assays (Fig. 7c, d). Immunofluorescence staining of cleaved caspase-3 further demonstrated that NSC-sEVs can attenuate apoptotic cell death in injured spinal cord (Supplementary Fig. S3).

### NSC-sEVs suppress the activation of microglia and reduce neuroinflammation after SCI

To evaluate the effect of NSC-sEVs on microglial activation after SCI, we quantified the numbers of CD68-positive (activated) microglia near the injury site by immunostaining. The number of CD68-positive microglia was substantially lower in the SCI + NSC-sEVs treatment group compared with the SCI group on the third day post-SCI (Fig. 8a, b). In addition, expression levels of the pro-inflammatory cytokines TNF-α, IL-1β, and IL-6 were significantly higher in SCI group rats than sham group and the SCI + NSC-sEVs group rats (Fig. 8c, d). Collectively, these data suggest that NSC-sEVs reduce neuroinflammatory responses following SCI.

### NSC-sEVs activate autophagy in vitro

As autophagy plays an important role in cytoprotection under stress, we examined the effect of NSC-sEVs on autophagy activation in spinal cord neurons. Glutamate can induce autophagy in nerve cells^40^. Primary neurons cultured with or without NSC-sEVs and then exposed to Glu exhibited autophagosomes as revealed by TEM. Compared with the Glu-only group and control group, however, NSC-sEVs + Glu group neurons demonstrated a significantly greater number of autophagosomes (Fig. 9a, b). To provide a more efficient method for quantitation of autophagy, we transfected spinal neurons with mRFP-GFP-LC3 virus and observed autophagy flux using laser confocal microscopy. Autophagosomes were labeled red and green (yellow fluorescence), whereas autophagic lysosomes were labeled red. The NSC-sEVs + Glu treatment group exhibited larger numbers of yellow and red fluorescent puncta than the Glu-only and untreated groups (Fig. 9c, d). We further evaluated autophagy using the Cyto-ID® Autophagy Detection Kit, and as expected, NSC-sEVs + Glu treatment increased autophagy levels compared with Glu alone (Fig. 9e, f). Moreover, western blotting results showed that NSC-sEVs + Glu treatment increased the expression levels of the autophagy-related proteins LC3BII and beclin-1, and decreased the level of P62 (Fig. 9g, h). Thus, NSC-sEVs can activate autophagy in spinal neurons in vitro.

### NSC-sEVs promote beclin-1 and LC3B expression after SCI

Immunofluorescence staining and western blot analyses of beclin-1 and LC3B expression were used to verify the effect of NSC-sEVs on autophagy in vivo. Beclin-1/NeuN/DAPI- and LC3B/NeuN/DAPI-positive cells were counted at the injury site under fluorescence microscopy as an index of autophagy activation in spinal neurons. Compared with the SCI group, the SCI + NSC-sEVs group demonstrated significantly greater numbers of beclin-1/NeuN/DAPI- and LC3B/NeuN/DAPI-positive cells, indicating that NSC-sEVs promote autophagy after SCI, consistent with our in vitro results (Fig. 10a–d). Western blot analysis confirmed the results of immunofluorescence analysis. Beclin-1 and LC3BII protein expression levels were significantly higher in the SCI + NSC-sEVs group than the SCI group, whereas p62 levels were lower than in the SCI group on the first day after injury (Fig. 10e, f).

### NSC-sEVs prevent apoptosis and secretion of inflammatory factors by activating autophagy

Considering the key role of autophagy in neuronal apoptosis and tissue inflammation^41,42^, we speculated that the reduced apoptosis and neuroinflammation observed following NSC-sEVs treatment depends on autophagy induction. To address this issue, primary neurons were treated with NSC-sEVs in the presence or absence of the autophagy inhibitor 3MA prior to Glu exposure. Consistent with our previous experiments, pretreatment with NSC-sEVs reduced the number of TUNEL-positive (apoptotic) neurons following Glu treatment compared with cultures treated with Glu alone (Fig. 11a, b). However, addition of the autophagy inhibitor 3MA during pretreatment reversed the anti-apoptotic effect of NSC-sEVs (Fig. 11a, b). Annexin V-FITC/PI double staining and flow cytometry results were consistent with these TUNEL results (Fig. 11c, d). Western blotting also indicated that 3MA inhibited elevation of the autophagy marker proteins LC3BII and beclin-1 and the anti-apoptotic protein BCL-2 induced by NSC-sEVs (Fig. 11f, g and j). Further, 3MA also reversed the reductions in Bax and cleaved caspase-3 expression observed following NSC-sEV treatment (Fig. 11h, i). Finally, 3MA pretreatment reversed the NSC-sEV-induced suppression of TNF-α, IL-1β, and IL-6 production by activated macrophages (Fig. 11k–m). Taken together, these results indicate that NSC-sEV-induced activation of autophagy is necessary for inhibition of apoptosis and neuroinflammatory responses.

## Discussion

Despite decades of intensive basic and clinical research into neurodegenerative and neuroprotective mechanisms, SCI still has generally poor prognosis^43^. Even neurons that survive the initial traumatic damage may be lost to ensuing pathogenic events such as neuroinflammation and apoptosis. This secondary damage leads to irreparable damage and loss of function^44^. Activation of microglia is a seminal early mediator of neuroinflammation and thus a major contributor to spinal damage and motor dysfunction^38^. In this study, we demonstrate for the first time that NSC-derived sEVs can suppress neuronal apoptosis, microglia activation, and neuroinflammation, thereby promoting functional recovery in SCI model rats. Further, these effects appear to depend on neuronal autophagy.

Stem cell transplantation is considered a promising potential treatment for central nervous system diseases given the capacity of stem cells to differentiate into multiple cell types^45,46^. Studies have shown that NSCs protect surviving neurons and promote functional recovery after SCI^47–49^ and hypoxia−ischemia by reducing inflammation^50–52^. However, the optimal treatment parameters are difficult to assess. If the NSCs are transplanted at high density, thrombosis will develop, but NSCs show relatively low survival rates in vivo^9^. Therefore, although NSC therapy has achieved some success in various animal disease models, many problems remain to be solved before clinical application.

SEVs are small vesicles released by cells that may contribute to cell−cell signaling by transmitting RNA, proteins, and bioactive lipids^53,54^. The origin of these vesicle is revealed by the specific pattern of surface antigens expressed^54,55^. sEVs produced by NSCs demonstrate therapeutic efficacy against ischemic, inflammatory, and neurodegenerative diseases^25,56,57^. sEVs may be superior for regenerative medicine because they circumvent many limitations of direct stem cell transplantation (e.g., low survival, de-differentiation, tumorigenesis)^58^. Moreover, stem cell transplantation therapy may work primarily through a paracrine mechanism involving sEVs^59,60^. Therefore, we hypothesized that direct administration of NSC-derived sEVs can overcome the limitations and challenges of direct stem cell therapy and promote functional recovery after experimental SCI.

Here, we conducted a series of experiments in vivo and in vitro to prove our hypothesis. First, we successfully extracted NSCs and isolated high concentrations of sEVs from the culture medium. sEVs with a diameter of 20−150 nm were then identified using DLS and further characterized by TEM and immunoblotting for the specific sEV surface markers CD9, CD63, and CD81. Pretreatment with these NSC-sEVs protected against glutamate excitotoxicity in vitro and secondary SCI in vivo, effects associated with suppression of neuroinflammation (microglial activation, NO release, and cytokine production) and promotion of autophagy. In fact, pharmacological experiments indicated that the anti-apoptotic and anti-inflammatory effects were directly dependent on activation of autophagy.

The pathogenic mechanisms of SCI are complex, but inflammation and apoptosis are the two major processes of secondary injury^44,61,62^. After neuronal injury, the expression levels of proapoptotic proteins Bax and cleaved caspase-3 are upregulated, whereas the expression of anti-apoptotic Bcl-2 is generally downregulated^63–65^. In vitro TUNEL staining, flow cytometry, and western blotting showed that pretreatment with NSC-sEVs reduced apoptosis of primary spinal neurons exposed to cytotoxic concentrations of Glu. To further confirm this anti-apoptotic mechanism, we evaluated the extent of apoptosis in the isolated spinal cord by TUNEL staining. As expected, in vivo TUNEL staining confirmed that NSC-sEVs can effectively prevent apoptosis of injured spinal cord neurons, whereas western blotting showed that expression levels of proapoptotic markers were downregulated and expression of the anti-apoptotic marker Bcl-2 was upregulated.

Inflammation following traumatic SCI involves activation of microglia and upregulation of neuroinflammatory cytokines such as TNF-α, IL-1β, and IL-6^38,61^. We found that LPS-induced NO production by isolated microglia was downregulated by pre-incubation with NSC-sEVs. In addition, the expression levels of pro-inflammatory cytokines were significantly suppressed by NSC-sEV pretreatment. Further, the number of CD68-positive (activated) microglia was significantly decreased in injured spinal cord pretreated with NSC-sEVs compared to untreated injured spinal cord, indicating that NSC-sEVs can reduce neuroinflammation in vivo as well as in vitro.

Autophagy is critical for regeneration. Many reports indicate that basal or physiological autophagy contributes to the maintenance of cellular homeostasis and in the quality control of proteins and subcellular organelles^31^. Pathological conditions or cellular stress can induce autophagy as an adaptive and protective mechanism^33^. Upregulation of LC3B and beclin-1 is a widely accepted marker for autophagy^66,67^. Studies in a rat model of traumatic brain injury have shown that autophagy can reduce cell damage^68^. Further, protective effects of autophagy have been confirmed in experimental models of traumatic SCI^42,69,70^. Baixauli et al.^71^ posited that sEVs in concert with the autophagy–lysosomal pathway maintain intracellular protein and RNA homeostasis. However, whether NSC-sEVs can activate target cell autophagy to prevent tissue damage has not been reported. Here, our in vitro TEM and mRFP-GFP-LC3 lentiviral transfection results showed that NSC-sEV pretreatment increased Glu-induced autophagy, findings confirmed by Cyto-ID imaging and western blotting of autophagy marker proteins. In vivo immunofluorescence experiments also demonstrated that NSC-sEV pretreatment can increase the expression of LC3B and beclin-1 at 6 and 24 h after SCI. To assess whether NSC-sEV-mediated autophagy is critical for inhibition of apoptosis and neuroinflammation, we co-treated spinal neurons with the autophagy-specific inhibitor 3MA and observed reversal of both the anti-apoptotic and anti-inflammatory effects of NSC-sEVs.

In summary, this study demonstrates for the first time that NSC-sEVs can effectively reduce neuronal apoptosis and neuroinflammation, thereby promoting functional recovery after SCI. In particular, we found that NSC-sEVs can suppress apoptosis and inflammatory processes by mediating autophagy. Taken together, these findings provide the basis for future use of sEVs as a new biological treatment for SCI.

## Supplementary information

Supplemental Figures

Supplementary figure legends
