# Supplementary figures and images for: Neural stem cell-derived small extracellular vesicles attenuate apoptosis and neuroinflammation after traumatic spinal cord injury by activating autophagy
Source: Cell Death Dis. 2019 Apr 18;10(5):340. doi: 10.1038/s41419-019-1571-8 (PMC6472377; doi:10.1038/s41419-019-1571-8)

## Supplementary figures

**Fig. S1**

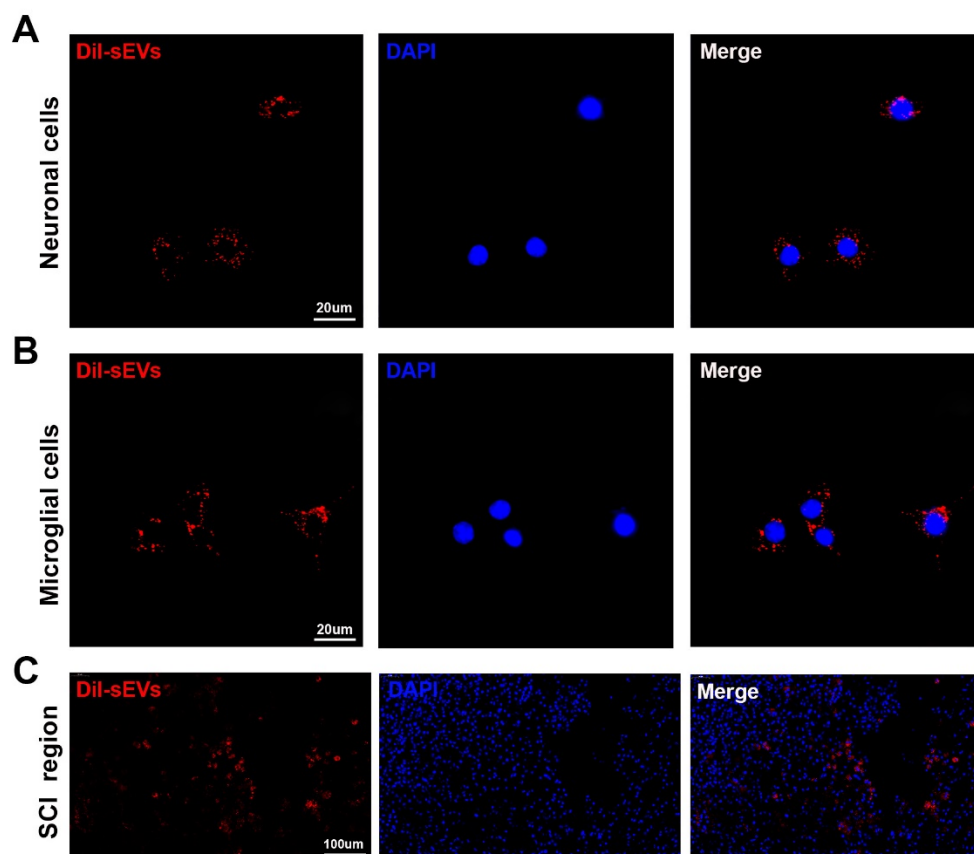

**Fig. S2**

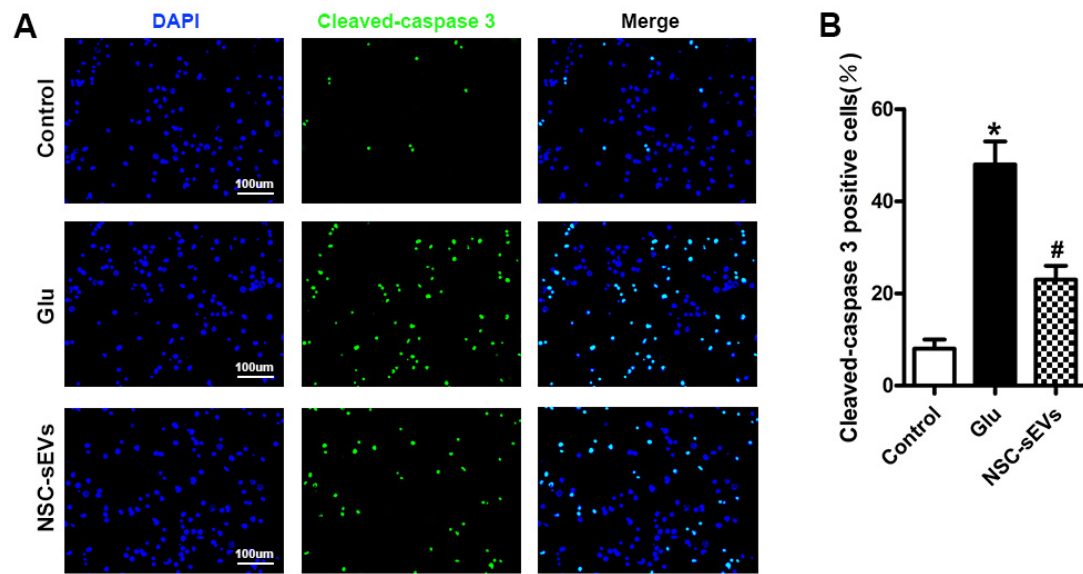

**Fig. S3**

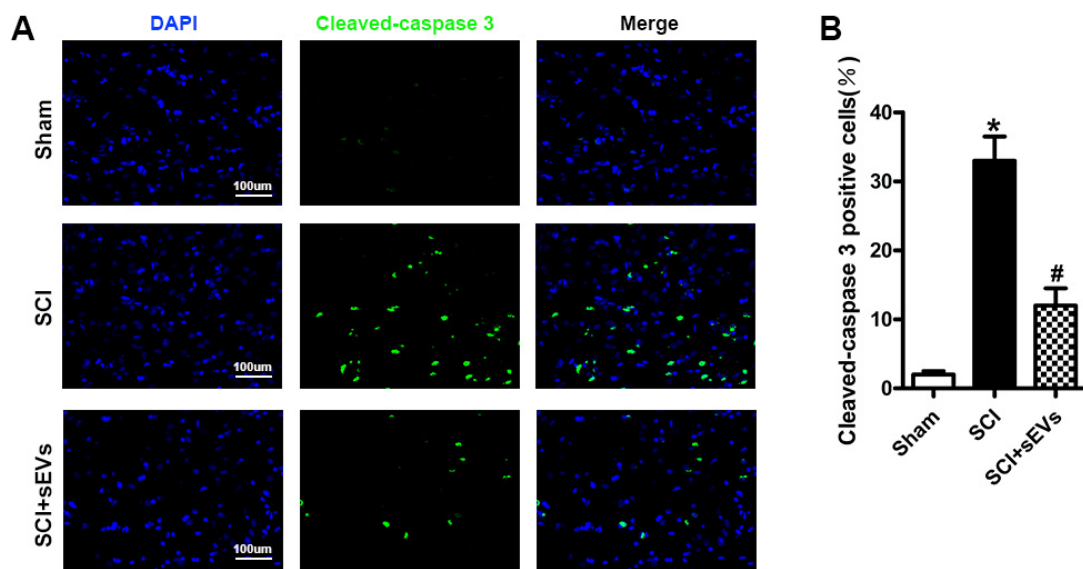

Supplement: Supplementary file 1 — Supplemental Figures [file 41419_2019_1571_MOESM1_ESM.pdf]
