# Supplementary material for: Neural stem cell-derived small extracellular vesicles attenuate apoptosis and neuroinflammation after traumatic spinal cord injury by activating autophagy
Source: Cell Death Dis. 2019 Apr 18;10(5):340. doi: 10.1038/s41419-019-1571-8 (PMC6472377; doi:10.1038/s41419-019-1571-8)
Supplement: Supplementary file 2 — Supplementary figure legends [file 41419_2019_1571_MOESM2_ESM.docx]

**Supplementary figure legends**

**Fig. S1 DiI-labeled NSC-sEVs uptake in vitro and in vivo. (A)** Uptake of DiI-labeled NSC-sEVs by primary cultured neurons. **(B)** Uptake of DiI-labeled NSC-sEVs by cultured microglia. **(C)** Uptake of DiI-labeled NSC-sEVs by cells at the spinal cord injury site. NSC-sEVs, neural stem cell-derived small extracellular vesicles.

**Fig. S2 Immunofluorescence detection of cleaved caspase-3 expression in glutamate-treated cells. (A)** Number of cleaved caspase-3-positive cells in the Glu + NSC-sEVs group was significantly lower than in the Glu alone group. **(B)** Quantification of cleaved caspase-3-positive cells in each experimental group. *p<0.05 compared to the Control group, #p<0.05 compared to the Glu group. NSC-sEVs, neural stem cell-derived small extracellular vesicles; Glu, glutamate.

**Fig. S3 Immunofluorescence detection of** **cleaved caspase-3 expression in spinal cord following injury. (A)** Numbers of cleaved caspase-3-positive cells in spinal sections as determined by fluorescence microscopy. The number of cleaved caspase-3-positive cells was significantly lower in the SCI + NSC-sEVs group than the SCI group. **(B)** Quantification of cleaved caspase-3-positive cells in each experimental group. *p<0.05 compared to the Control group, #p<0.05 compared to the Glu group. NSC-sEVs, neural stem cell-derived small extracellular vesicles.
